# An independently reconfigurable dual-mode dual-band substrate integrated waveguide filter

**DOI:** 10.1371/journal.pone.0179816

**Published:** 2017-06-23

**Authors:** Yongle Wu, Yuqing Chen, Lidan Yao, Weimin Wang, Yuanan Liu

**Affiliations:** Beijing Key Laboratory of Work Safety Intelligent Monitoring, School of Electronic Engineering, Beijing University of Posts and Telecommunications, Beijing, China; Ludwig-Maximilians-Universitat Munchen, GERMANY

## Abstract

In this paper, a novel perturbation approach for implementing the independently reconfigurable dual-mode dual-band substrate integrated waveguide (SIW) filter is proposed. Dual-frequency manipulation is achieved by adding perturbation via-holes (the first variable) and changing the lengths of the interference slot (the second variable) in each cavity. The independent control of the upper passband only depends on the second variable while the lower passband is independently tuned by combining the two variables. Using such a design method, a two-cavity dual-band SIW filter is designed and experimentally assessed with four via-holes and an interference slot in each cavity. The dual-band filter not only has a frequency ratio (*f*_R_) ranging from 1.14 to 1.58 but also can be considered as a single passband one with a tunable range of 40.5% from 1.26 GHz to 2.12 GHz. The scattering parameters |*S*_11_| and |*S*_21_| are in the range of -10.72 dB to -37.17 dB and -3.67 dB to -7.22 dB in the operating dual bands, respectively. All the simulated and measured results show an acceptable agreement with the predicted data.

## Introduction

Multiband bandpass filters are of interest in many wireless applications for interference reduction in a two-way radio system, different frequency bands utilization in the congested spectrum of electromagnetic waves, and compatibility of wireless devices with different standards. Normally, integrating a bank of fixed-frequency filters in a wireless system will definitely add to the complexity. This factor inspires developing filters that can not only simultaneously work at multiple bands but also dynamically tune the operating bands if needed. Recent advancements in filter technology, tunable/reconfigurable bandpass filters have gained a remarkable research interest with reducing complexity/loss in signal routing and meeting the present requirements of system size, complexity and cost reduction.

Recently, some traditional materials are used to implement filters through different theoretical techniques. Besides, there are new materials which can be operated at microwave frequency band such as graphene [1–2] and topological insulator: Bi2Te3 [3]. Unlike traditional substrates with metal plated on its surface, the topological insulator achieves conductivity by itself, while the devices about the topological insulator lack practical demonstrations. So far a few of tunable/reconfigurable filters with various materials and technologies have been realized to satisfy the increasing demands in multiband transceiver architectures for modern multistandard communication systems. The filters are mainly designed in these theories including graphene [2], substrate integrated waveguide (SIW) [4–7], coplanar waveguide (CPW) [8–9], microstrip [10–12], and cavity [13–14]. In [2, 4, 5, 8, 9, 13], tunable passband filters are all designed based on a passband. Thus they cannot be operated at multiple passband simultaneously. The filters with two passbands in [6] and [10] are generated by dual modes, the lower passband can be reconfigurable without affecting the other one, but the upper passband cannot be tuned independently. Excitedly, with the continuous exploration of the researchers, in [7], [11–12] and [14], the lower and upper passbands of the designed filters can be tuned and are relatively independent of each other. Due to the advantages of the SIW technology such as easy fabrication, simple integration with active devices, higher quality factor, a few of SIW filters with single passband tunable have been proposed. However, few SIW filters with dual bands tuned independently have been put forward.

In this paper, we propose a novel simple approach, adding perturbation via-holes and changing the lengths of the interference slots, for designing a single-layer dual-mode dual-band independently reconfigurable SIW filter. The approach, operating the electromagnetic distributions of one of two resonant modes (the TE_101_ and the TE_102_ modes) of the structure, are exploited to change its resonant frequency. In the following sections we will first give the traditional theoretical design of dual-mode dual-band SIW filter, and the influences of electromagnetic distributions of two resonate modes are analyzed for different disturbances on every original SIW structure. Second, put forward a new perturbation method and a set of dual-mode dual-band SIW structures with different numbers of via-holes and lengths of the interference slots are applied to validate the design methodology. Finally, the dual-mode dual-band SIW filter with four via-holes and a fixed size of interference slot on each cavity is presented. The upper passband can be tuned individually only depending on the changed lengths of the interference slots, while the independent control of the lower passband needs to be combined the changed lengths of the interference slots and different numbers of via-holes. It can be found that this proposed SIW filter has a number of attractive features, which are: 1) flexible independently-reconfigurable dual bands; 2) simple via-holes and slots without any complex components; 3) easy integration with single-layer structure; 4) low-cost fabrication using the printed circuit board technology.

## Methods

### Theoretical design and modes analysis

**Fig 1:**
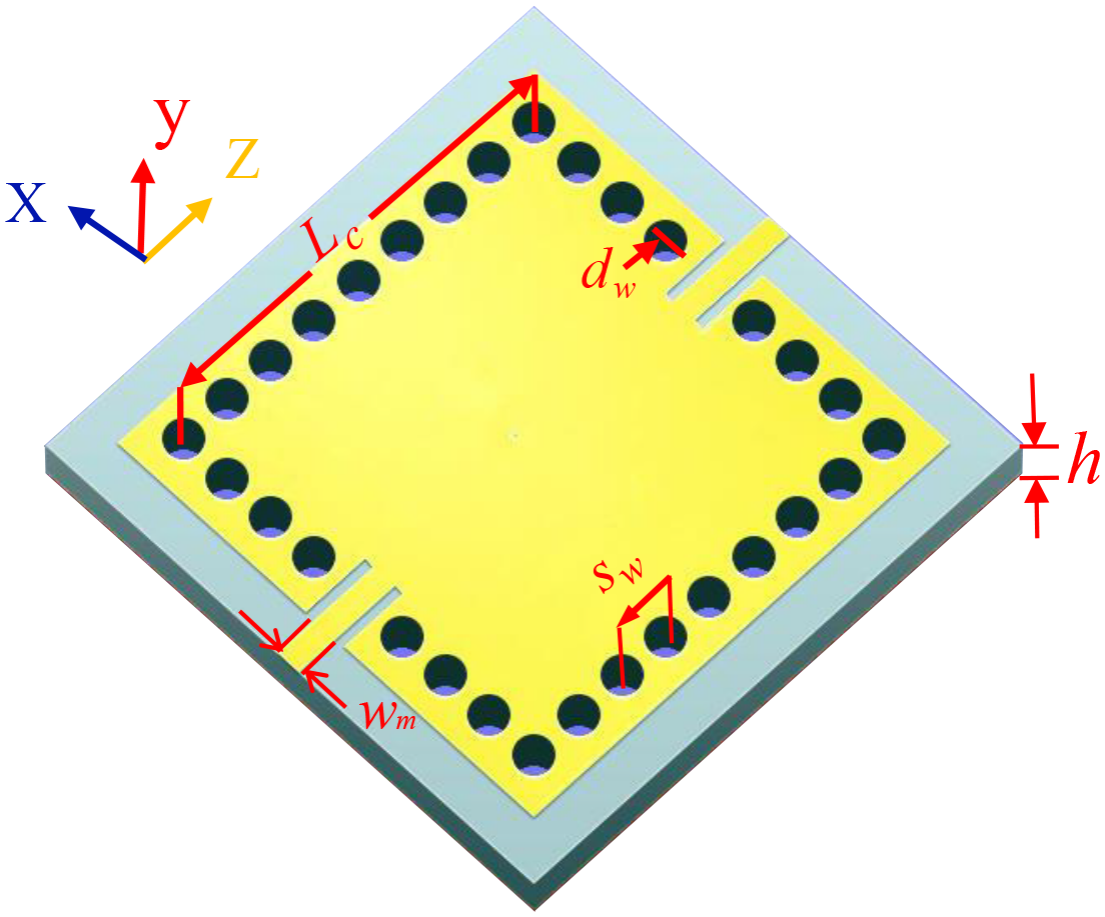
A square SIW cavity without any interference. The whole bottom and the top yellow area of the cavity is constructed by metal. *L*_c_ is the length of the square SIW cavity, *d*_w_ is the diameter of metallized via holes, *s*_w_ is the center spacing between two adjacent metallized via holes. This SIW cavity is excited by two 50 Ω microstrip feeding lines, *h* is the thickness of a used substrate, *w*_m_ is the width of the microstrip line. It is necessary to take appropriate values for *s*_w_, *d*_w_, and *L*_c_ to avoid dispersion loss of typical SIW structure.

Fig 1 illustrates the geometrical structure of a single square SIW cavity directly excited by two 50 Ω microstrip feeding lines and the bottom of the used substrate is constructed completely by a metal. *w*_m_ is the width of the microstrip feeding lines and *h* is the thickness of the chosen substrate. *L*_c_ is the length of the single square cavity. *d*_w_ is the diameter of metallized via holes. *s*_w_ is the center spacing between two adjacent metallized via holes. To achieve dual-mode dual-band SIW filter with the lower and the upper frequency generated by the TE_101_ and the TE_102_ modes, respectively, the initial sizes in Fig 1 can be calculated by the following equation [15]:

$$f_{m0n}=\frac{c_{0}}{2\sqrt{\varepsilon_{r}}}\sqrt{\frac{m^{2}+n^{2}}{{\left(L_{c}-\frac{d_{\text{w}}{}^{2}}{0.95s_{\text{w}}}\right)}^{2}}}\;$$ (1)

Where *m* and *n* are the half-wave numbers propagating along the x-axis and the z-axis, respectively, *f*_101_ and *f*_102_ are the resonant frequencies of the TE_101_ and the TE_102_ modes, respectively, *c*_0_ is the light velocity in vacuum, *ε*_r_ is the relative dielectric constant of the chosen substrate. Note that the following conditions [16] should be considered to avoid dispersion loss of typical SIW structures:

$$\frac{s_{\text{w}}}{d_{\text{w}}}<2.0,\;\frac{d_{\text{w}}}{L_{\text{c}}}<\frac{1}{5}$$ (2)

$$\frac{s_{\text{w}}}{d_{\text{w}}}<2.5,\;\frac{d_{\text{w}}}{L_{\text{c}}}<\frac{1}{8}\;$$ (3)

When *d*_w_/*L*_c_ increases, a smaller *s*_w_/*d*_w_ is needed.

**Fig 2:**
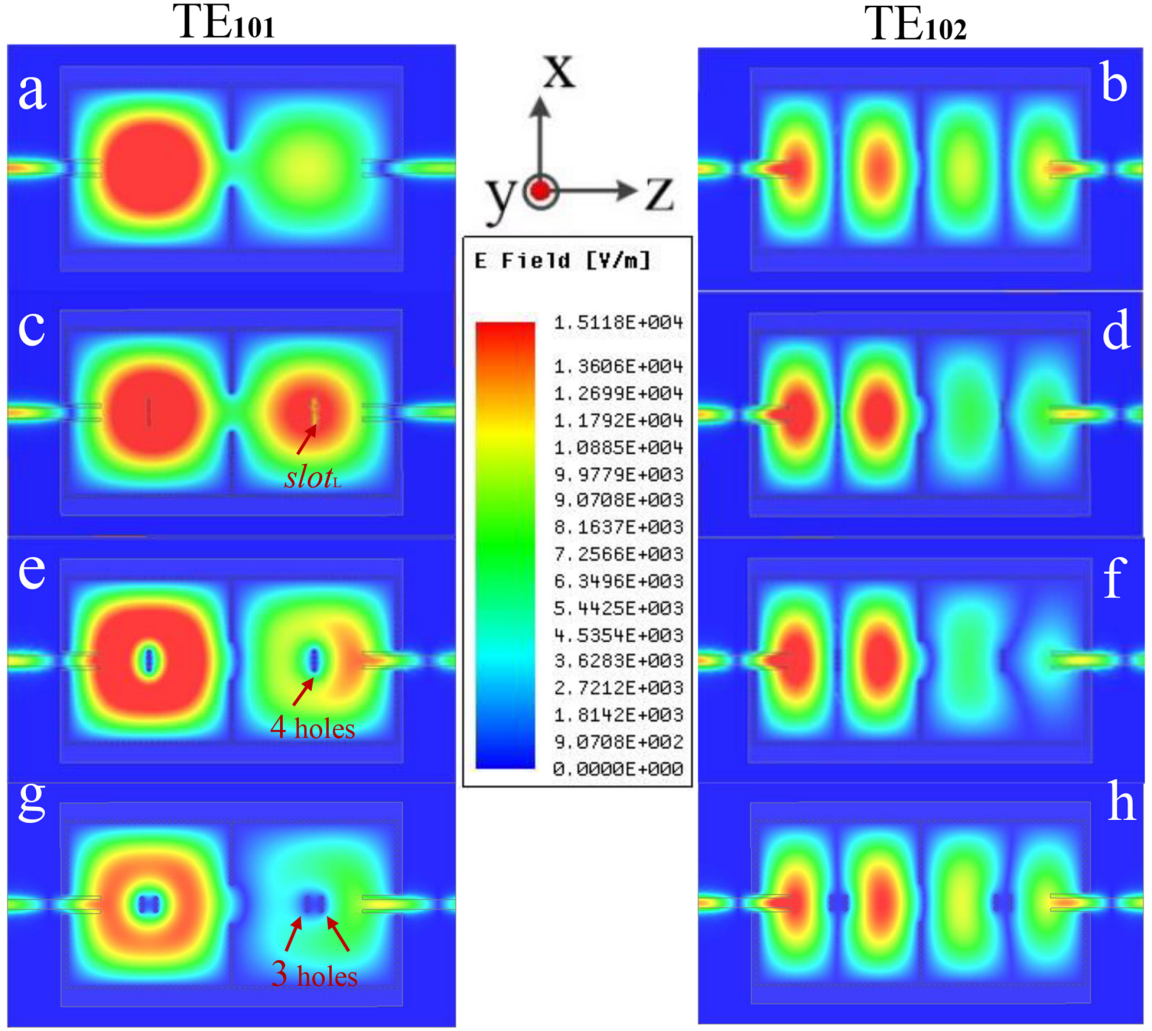
The electromagnetic distributions of the TE_101_ and the TE_102_ modes in different perturbation states. The SIW filter is composed of two square cavities in Fig 1, (a-b) The two square cavities without any interference, (c-d) An interference slot with the length of *slot*_L_ on the vertical line of each square cavity, (e-f) Four perturbation via-holes on the vertical line of each cavity, (g-h) Three perturbation via-holes on each side of the vertical line of each square cavity.

The electromagnetic distributions of different perturbation in a dual-mode dual-band SIW filter are illustrated in Fig 2. The simulated substrate is Rogers 4350B with relative permittivity of 3.48, thickness *h* of 0.762 mm and loss tangent of 0.0037. *d*_p_ is the diameter of perturbation via holes. *s*_p_ is the distance between the center positions of two adjacent perturbation via holes. *slot*_L_ (*slot*_w_) is the length (width) of interference slots. The desired dimensions are: *L*_c_ = 28.4 mm, *w*_m_ = 1.72 mm, *d*_w_ = 0.6 mm, *s*_w_ = 1 mm, *d*_p_ = 0.6 mm, *s*_p_ = 1 mm, *slot*_L_ = 5 mm and *slot*_w_ = 1 mm. Fig 2a and 2b show the electromagnetic distributions of the dual-mode dual-band SIW filter without any perturbation. The electromagnetic distributions of Fig 2c and 2d originate from the dual-mode dual-band SIW filter [17] with an interference slot on the vertical line of each cavity, and Fig 2e and 2f originate from the dual-mode dual-band SIW filter [6] with four perturbation via-holes on the vertical line of each cavity.

Comparing Fig 2c–2f with Fig 2a and 2b, an additional interference slot in the middle of each cavity just influences the electromagnetic distribution of the TE_102_ mode, but does not affect the electromagnetic distribution of the TE_101_ mode. However, changing the interference slot to four perturbation via-holes in the same place, the situation is exactly the opposite. Despite that the TE_102_ and the TE_201_ modes are degenerate modes in a square cavity, the TE_201_ mode is suppressed by the additional interference slot and perturbation via-holes.

The foregoing conclusions have been drawn from the papers [6] and [17]. Based on the conclusions, an inspiration is obtained that combining the additional interference slots and perturbation via-holes to achieve the tunable dual bands independently. However, there still exists an unsolved problem that it is difficult to achieve tunable dual bands independently when both perturbation via-holes and an interference slot are in the middle of the cavity at the same time. Thus the locations of the perturbation via-holes are changed symmetrically on both sides of the vertical bisector of each cavity. In contrast with Fig 2a and 2b, the perturbation via-holes symmetrically on both sides of the vertical bisector of each cavity have effects on the electromagnetic distributions of the TE_101_ and the TE_102_ modes in Fig 2g and 2h. In addition, note that the influence on the TE_101_ mode is greater than that on the TE_102_ mode in Fig 2g and 2h.

In order to achieve independent controllable dual bands, a new way of interference needs to be found, which only affects the electromagnetic distribution of the TE_101_ mode without affecting that of the TE_102_ mode. Comparing Fig 2d with Fig 2h, it is found that the effects of the perturbation via-holes and an interference slot on the electromagnetic distribution of the TE_102_ mode are reversed. Therefore, the effects of the specific number of perturbation via-holes and the specific length of an interference slot on the electromagnetic distribution of the TE_102_ mode can be offset from each other. Besides, an interference slot has no influence on the electromagnetic distribution of the TE_101_ mode. Therefore, the upper frequency generated by the resonate mode TE_102_ will not be affected. Then the lower passband generated by the resonate mode TE_101_ can be independently tuned by combining different additional numbers of perturbation via-holes and different lengths of the interference slots, and the upper passband can be independently tuned only by changing the lengths of the interference slots.

**Fig 3:**
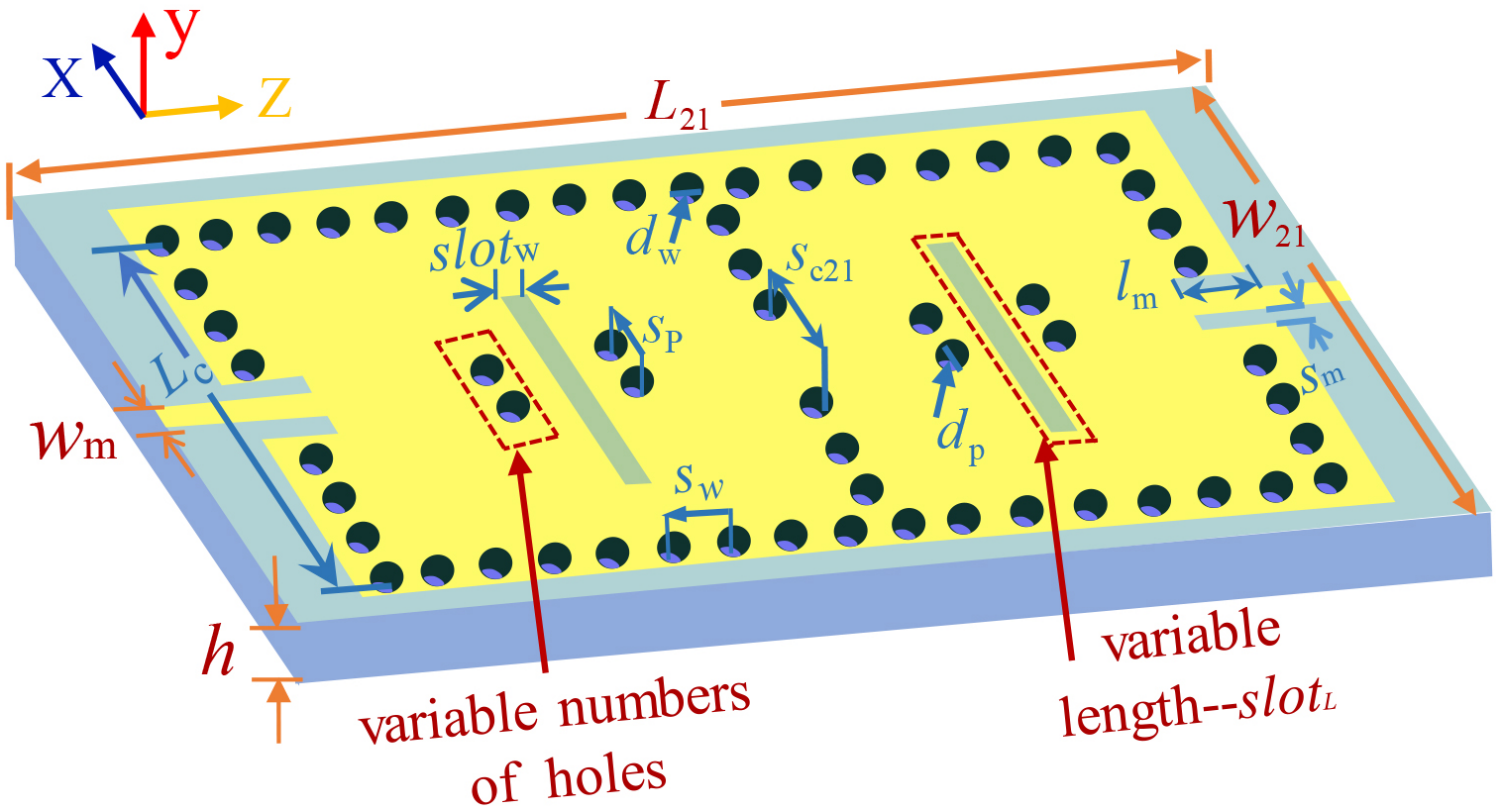
A dual-band SIW filter with four perturbation via-holes and an interference slot in each cavity. The used substrate and the dimension are the same as the ones in Fig 2. *L*_21_ = 77 mm, *w*_21_ = 44 mm. In the following section, some filters will be needed, with the shapes and sizes of which are the same as the ones in Fig 3, except for the number of holes and the variable length (*slot*_*L*_). In order to avoid presenting several similar graphs, they are all shown in Fig 3, and the variable numbers of holes and the variable length are used in Fig 3 to differentiate them. The remaining variables (*l*_m_, *s*_m_, and *s*_c21_) are related to the number of perturbation via-holes and the length of the interference slot in each cavity.

**Fig 4:**
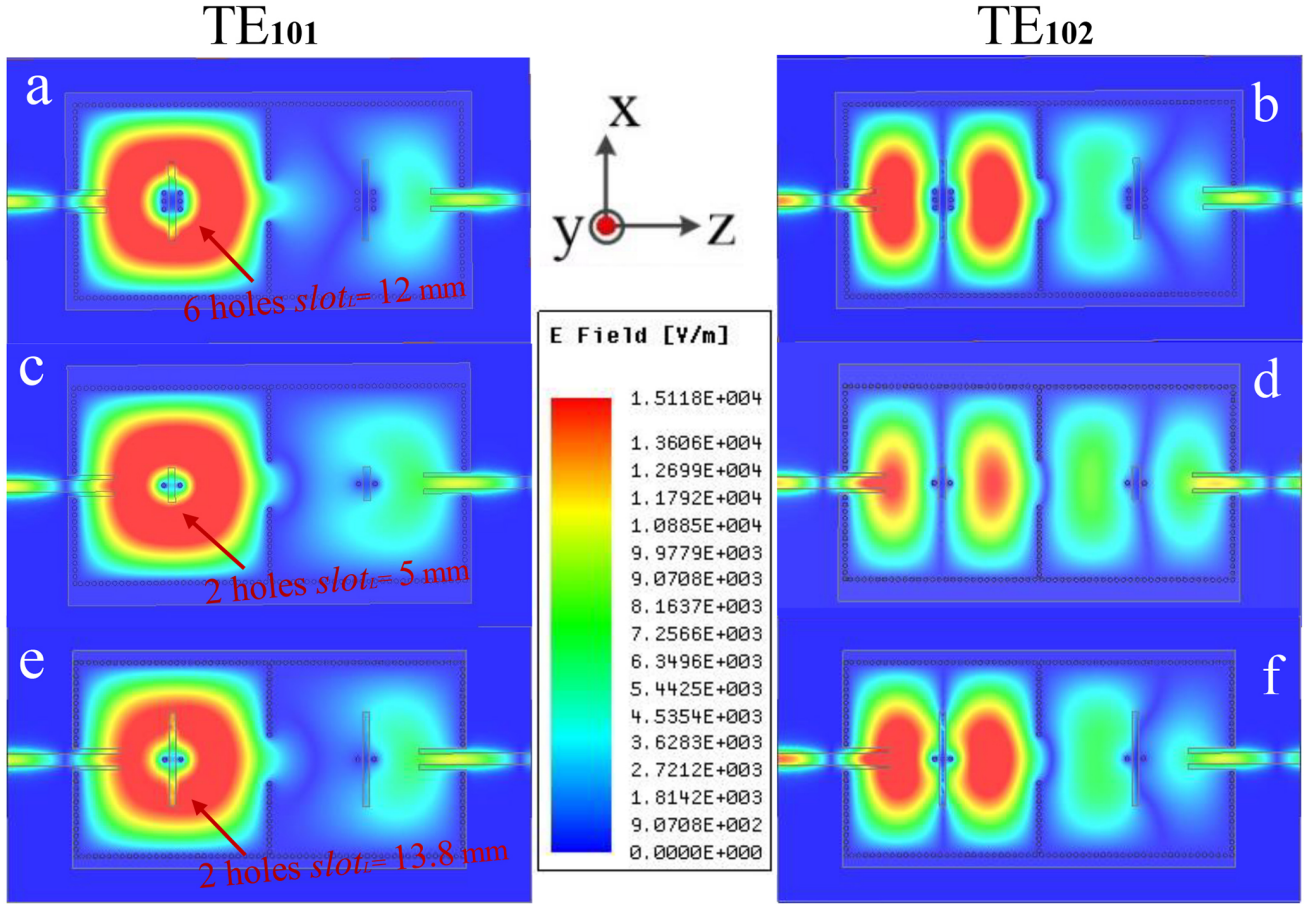
The electromagnetic distributions of the dual-band SIW filter with the different perturbations. (a-b) Six perturbation via-holes and *slot*_*L*_ = 12.0 mm in each cavity. (c-d) Two perturbation via-holes and *slot*_*L*_ = 5.0 mm in each cavity. (e-f) Two perturbation via-holes and *slot*_*L*_ = 13.8 mm in each cavity.

**Fig 5:**
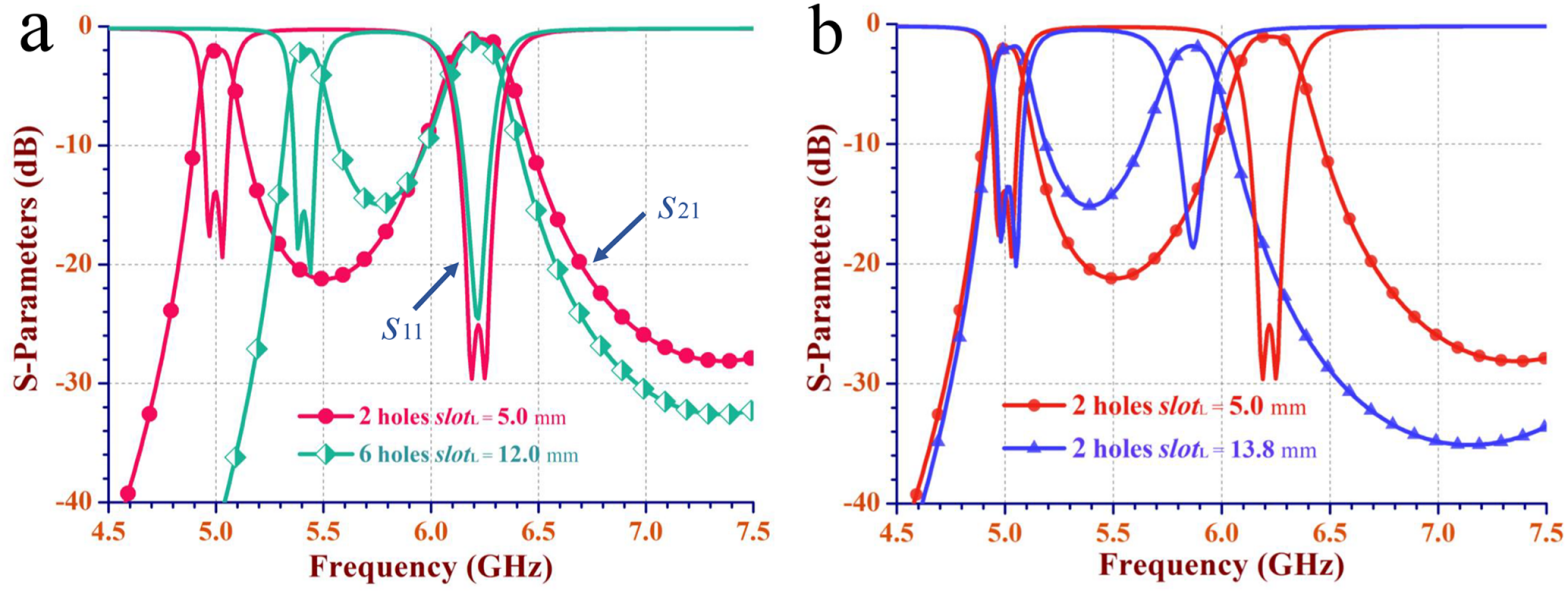
The simulated scattering-parameters of the dual-band SIW filter in Fig 4. (a) The simulated scattering parameters (*S*_11_ and *S*_21_) correspond to the electromagnetic distributions in Fig 4a–4d. (b) The simulated scattering parameters correspond to the electromagnetic distributions in Fig 4c and 4f.

Inspired by the analysis and comparison of electromagnetic distributions in Fig 2, a two-cavity dual-band SIW filter with four perturbation via-holes and an interference slot in each cavity is showed in Fig 3. An interference slot is on the vertical bisector of the z axis in each cavity. Besides, the four holes are symmetrically distributed on both sides of the interference slot. The corresponding electromagnetic distributions are presented in Fig 4. In contrast with Fig 4a and 4c shows a smaller range of interference to the electromagnetic distribution of the TE_101_ mode in the red area. Nevertheless, Fig 4c and 4e demonstrate a similar range of interferences to the electromagnetic distributions of the TE_101_ mode in the red areas. Thus, the lower frequencies generated by the TE_101_ mode in Fig 4c and 4e will be the same, and both these two frequencies in Fig 4c and 4e will be different from the one in Fig 4a. Compared to the electromagnetic distribution of the red area in Fig 4f, it can be observed that only the green areas are affected in Fig 4b and 4d. Even if the scopes of influence in green areas are different, the upper frequencies generated by the TE_102_ mode in Fig 4b and 4d will be the same due to the relatively weaker electromagnetic energy in the green areas. The corresponding simulated scattering-parameters are illustrated in Fig 5.

Fig 5a shows the simulated scattering parameters with the different fixed perturbation via-holes and lengths of the interference slot. Obviously, the frequency of lower-passband shifts from 5.01 GHz to 5.41 GHz, while the frequency of upper passband keeps at 6.22 GHz. Fig 5b shows the simulated scattering parameters with the same fixed perturbation via-holes and different lengths of the interference slot. Evidently, the frequency of upper passband shifts from 5.87 GHz to 6.22 GHz, while the frequency of lower passband maintains at 5.01 GHz.

**Fig 6:**
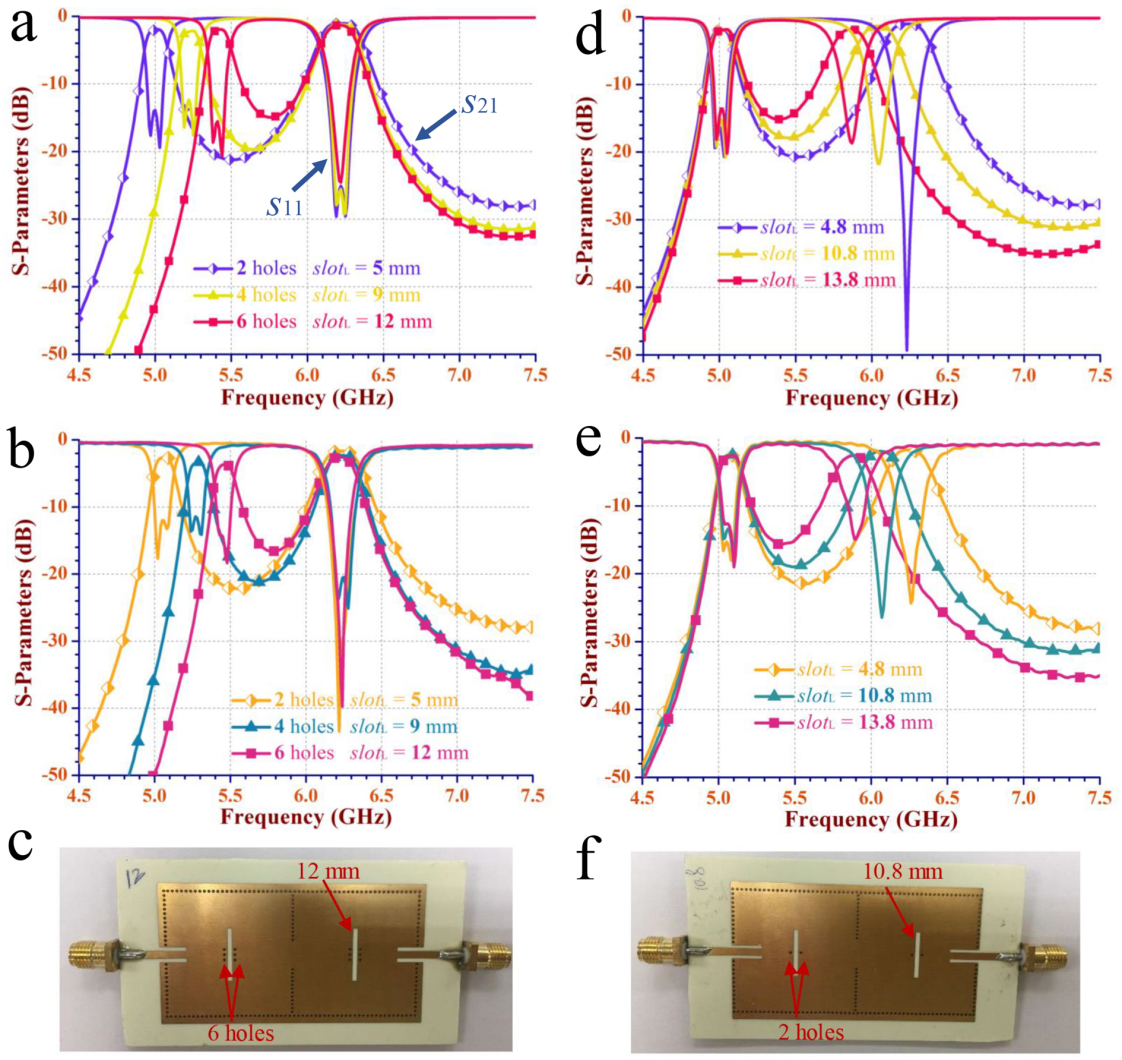
The simulated and measured scattering-parameters of the dual-band SIW filter in Fig 3. (a-b) The simulated and measured scattering-parameters (*S*_11_ and *S*_21_) of the dual-mode dual-band SIW filter in Fig 3 with two different interference variables, the number of via-holes and the lengths of the interference slots, respectively. (c) The fabricated PCB of the dual-mode dual-band SIW filter with six via-holes and *slot*_*L*_ = 12.0 mm in each cavity. (d-e) The simulated and measured scattering-parameters of the dual-mode dual-band SIW filter in Fig 3 with two via-holes and different lengths of the interference slots, respectively. (f) The fabricated PCB of the dual-mode dual-band SIW filter with two via-holes and *slot*_*L*_ = 10.8 mm in each cavity.

Fig 6a and 6b demonstrate the simulated and measured scattering-parameters of the dual-mode dual-band SIW filter in Fig 3 with different numbers of perturbation via-holes and lengths of the interference slot in each cavity, respectively. One of the related fabricated PCBs is displayed in Fig 6c. Fig 6d and 6e illustrate the simulated and measured scattering-parameters of the dual-mode dual-band SIW filter in Fig 3 with two perturbation via-holes and different lengths of the interference slot in each cavity, respectively. One of the related fabricated PCBs is displayed in Fig 6f. The substrates of all the fabricated PCBs are the same as the simulated ones in Fig 2, except that the metal of the simulated substrates are all replaced by 1/2oz thick copper in measured ones.

When the number of perturbation holes and the length of the interference slot increase from 2 to 6 and 5 mm to 12 mm, respectively, the lower frequency shifts from 5.05 GHz to 5.48 GHz, meanwhile, the upper frequency keeps at 6.22 GHz. When the number of perturbation via-holes maintains 2 and the length of the interference slot increases from 4.8 mm to 13.8 mm, the upper frequency shifts from 6.26 GHz to 5.90 GHz, meanwhile, the lower frequency keeps at 5.06 GHz. Obviously, to control the lower resonant frequency shift individually, the length of the interference slot grows longer as the number of perturbation via-holes increases. To independently tune the upper resonant frequency, only the length of the interference slot needs to be changed.

## Results

**Fig 7:**
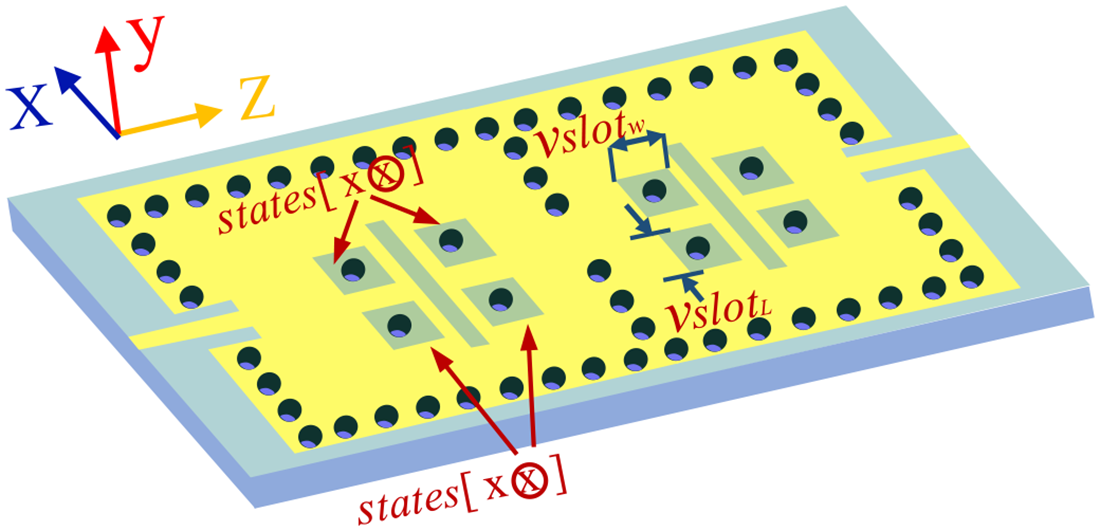
The SIW filter with four perturbation holes surrounded by the connected slots in each cavity. The states of the perturbation holes corresponding to x are the same. The value of x is 1 when the corresponding perturbation holes are connected to the top metal layer, and vice versa.

The lower frequency can be tuned independently on condition that the perturbation holes are variable. Hence, to prevent perturbing holes linking the top metal layer directly, four perturbing holes of each cavity in Fig 7 are surrounded by four connected slot in the top layer, respectively. The used substrate in Fig 7 is CER-10 with relative permittivity of 9.5, thickness *h* of 0.64 mm and loss tangent of 0.0035. The desired dimensions are: *L*_c_ = 55.4 mm, *L*21 = 125.0 mm, *w*_m_ = 0.63 mm, *w*_21_ = 61.6 mm, *d*_w_ = 1.0 mm, *s*_w_ = 2.0 mm, *d*_p_ = 0.6 mm, *s*_p_ = 4.5 mm, *l*_m_ = 12.85 mm, *s*_m_ = 3.24 mm, and *s*_c21_ = 11.49 mm.

However, the actual production may lead to a slight difference in the length (*Vslot*_L_), width (*Vslot*_w_) of the connected slots and the width (*slot*_w_) of the interference slots. The interference slot at perpendicular bisector located on the z-axis in each cavity is equivalent to open-circuited perturbation and then the changes in *slot*_w_ may result in shifts in the upper frequency. In Fig 2, the perturbation via holes symmetrically on both sides of the vertical bisector of each cavity influence the electromagnetic distributions of the TE_101_ and TE_102_ modes. We need to measure whether a small range of variations in *Vslot*_L_, *Vslot*_w_ and *slot*_w_ cause a shift in the two passband frequencies.

**Fig 8:**
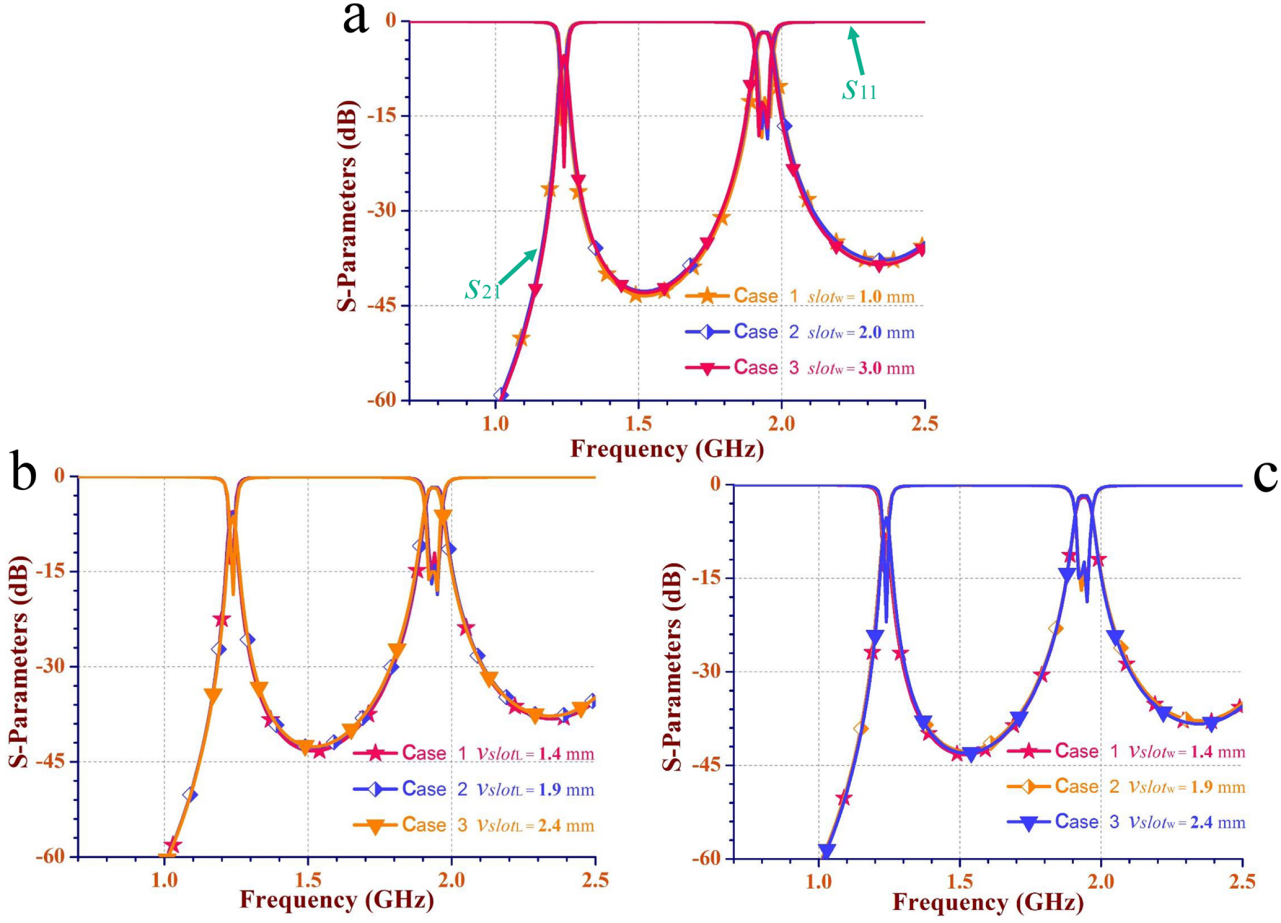
The sensitivity analysis for the SIW filter in Fig 7. (a) The scattering parameters (*S*_11_ and *S*_21_) of the interference slot with different widths (*slot*_w_) in each cavity. (b-c) The scattering parameters of a connected slot with different lengths (*Vslot*_L_) and widths (*Vslot*_w_) in each cavity, respectively.

Fig 8a–8c depict the return loss (*S*_11_) and the insert loss (*S*_21_) of the filter with variable *slot*_w_, *Vslot*_L_ and *Vslot*_w_, respectively. *slot*_w_1 = 1.0 mm, *slot*_w_2 = 2.0 mm, and *slot*_w_3 = 3.0 mm. *Vslot*_L_1 and *Vslot*_w_1 = 1.4 mm, *Vslot*_L_2 and *Vslot*_w_2 = 1.9 mm, *Vslot*_L_3 and *Vslot*_w_3 = 2.4 mm. When *slot*_w_ varies from 1.0 mm to 3.0 mm, the lower and the upper frequencies shift 0.81% and 0.52%, respectively. When *Vslot*_L_ or *Vslot*_w_ varies from 1.4 mm to 2.4 mm, the lower frequency shifts 0.81%, while little had altered in the upper frequency. The deviations are considered acceptable. Nevertheless, the variations impact on the return loss and insert loss. The maximum difference in return loss and insert loss of the dual passbands are 7.2 dB and 0.6 dB, respectively.

**Fig 9:**
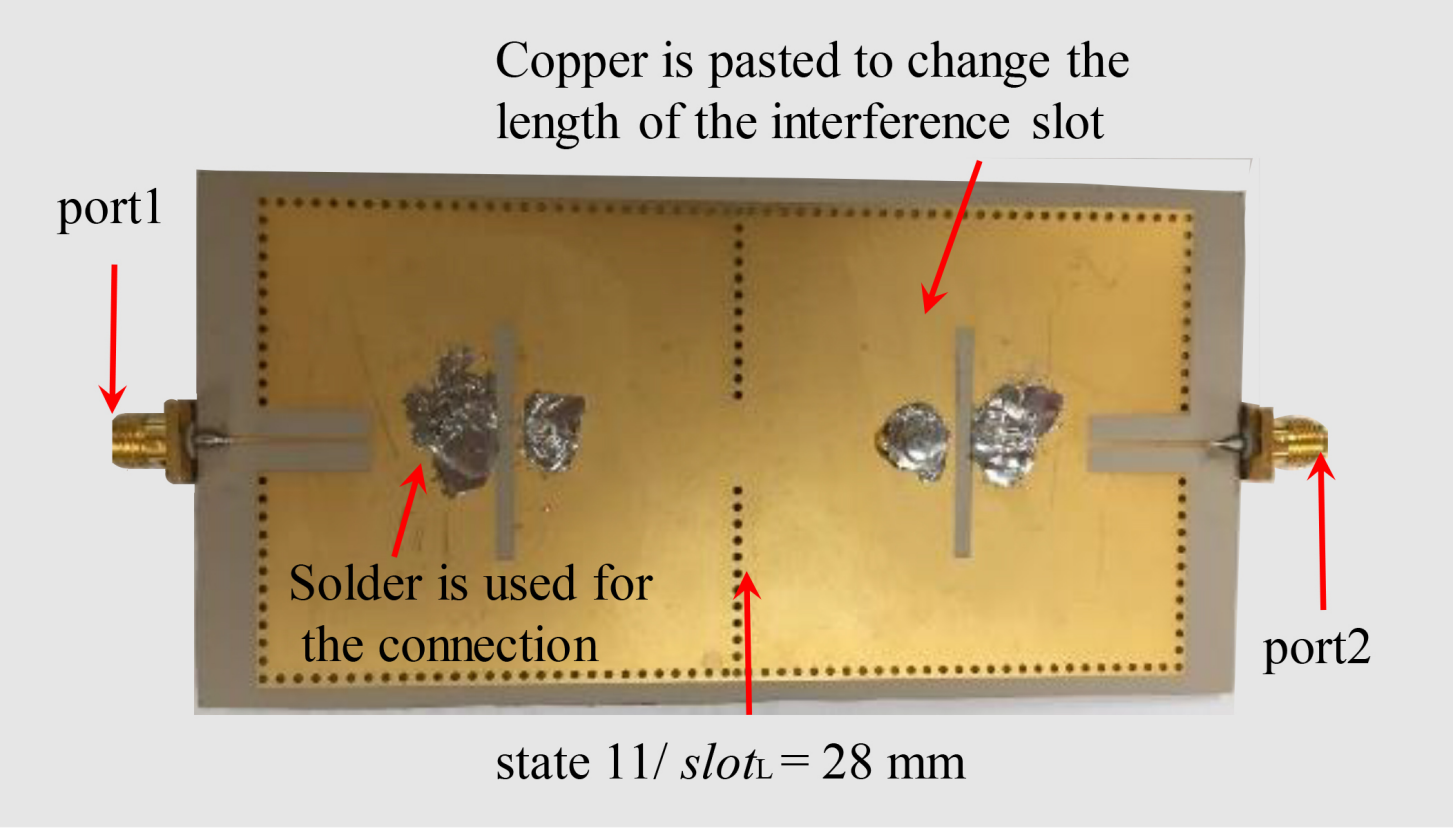
The photograph of the proposed SIW filter PCB. The experiment contains different states and only one state (state 11/ *slot*_L_ = 28 mm) is showed in Fig 9. The interference slot length (*slot*_L_) of the SIW filter is 28.0 mm. The changes in the interference slot length are achieved by copper. state 11 means all perturbation holes are connected to the top metal layer by solder.

Fig 9 shows the fabricated PCB of the dual-mode dual-band independently reconfigurable SIW filter depicted in Fig 7 with the dimension of 125×61.6 mm^2^. *slot*_w_ = 2.0 mm, *Vslot*_w_ = 1.9 mm, and *Vslot*_L_ = 1.9 mm. The substrate of the fabricated PCB is the same as the simulated one in Fig 7, except that the metal of the simulated substrate is replaced by 0.3 μm thick gold in measured one. The connections of the perturbation holes to the top metal layer are achieved by solder and the changes in the length of the interference slots are realized by pasting copper. Note that ‘0’ and ‘1’ are considered to be disconnected and connected with perturbation holes, respectively.

**Fig 10:**
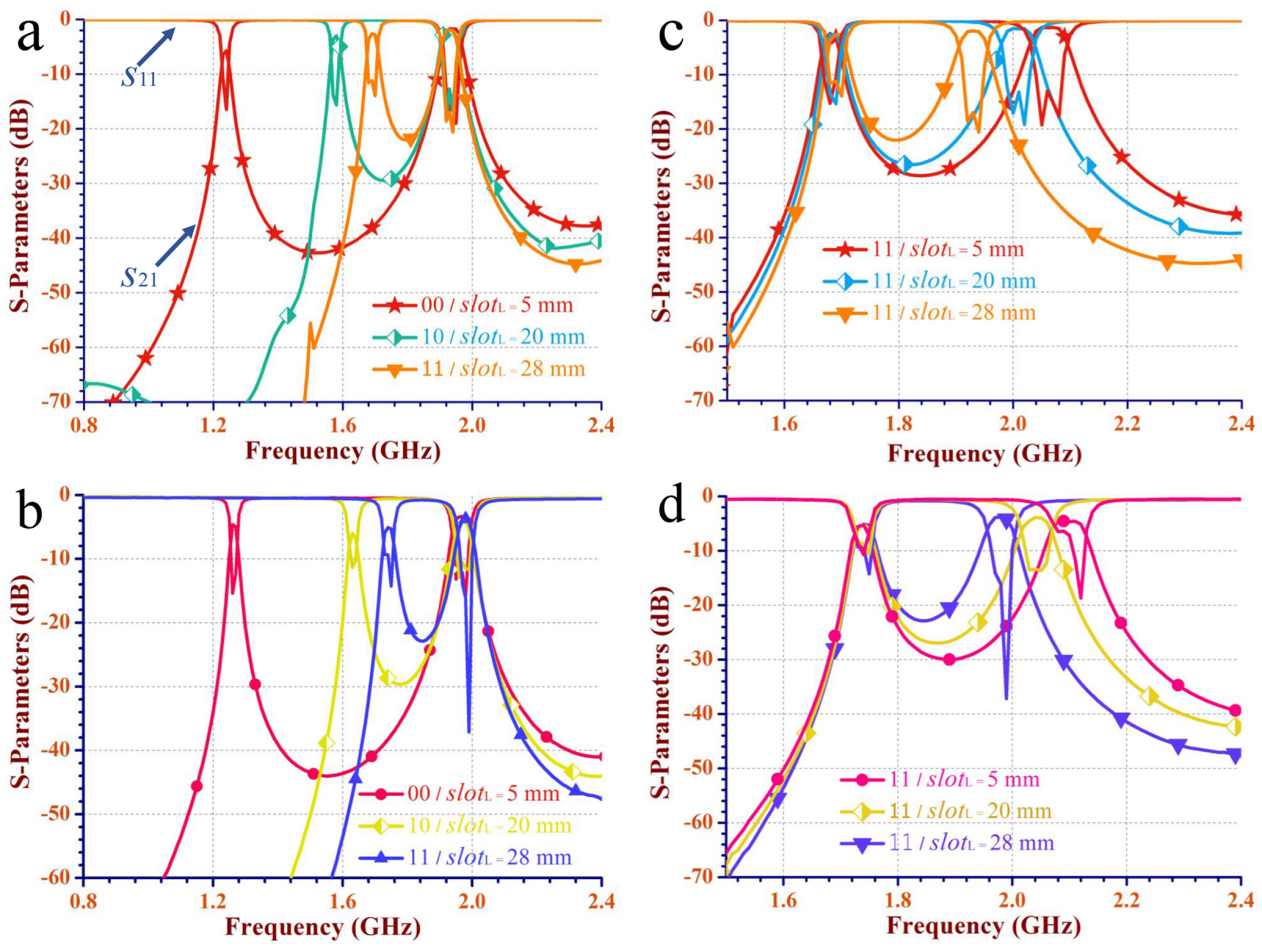
The simulated and measured scattering-parameters of the proposed SIW filter in Fig 9. (a) and (c), The simulated scattering parameters (*S*_11_ and *S*_21_). (b) and (d), The measured scattering parameters.

Fig 10a and 10c illustrate the simulated scattering parameters of the dual-mode dual-band independently reconfigurable SIW filter in Fig 9. Besides, Fig 10b and 10d illustrate the corresponding measured scattering parameters. Fig 10a and 10b show that the lower frequency is tunable while the upper frequency is unchanging. Fig 10c and 10d show that the upper frequency is tunable while the lower frequency is constant.

When the state varies from 00 to 11, and the corresponding length of the interference slot (*slot*_L_) varies from 5.0 mm to 28 mm, the lower frequency (the resonant frequency of the TE_101_ mode) is tuned with a range of 29.1% from 1.26 GHz to 1.75 GHz. Whereas the upper frequency (the resonant frequency of the TE_102_ mode) has an offset of 0.50% at around 1.99 GHz. When the state keeps for 11 with the length of the interference slot (*slot*_L_) varying from 5.0 mm to 28 mm, the upper frequency is tuned from 1.99 GHz to 2.12 GHz with a range of 6.5%, while the lower frequency is at around 1.75 GHz with an offset of 0.57%. It is shown that *f*_R_ ranges from 1.14 to 1.58.

Note that the state 11 and *slot*_L_ = 28 mm are both applied in Fig 10b and 10d, thus, the independently reconfigurable dual-band SIW filter can be considered as a single passband one with a tunable range of 50.9% from 1.26 GHz to 2.12 GHz. The return loss |*S*_11_| and the insert loss |*S*_21_| are in the range of -10.72 dB to -37.17 dB and -3.67 dB to -7.22dB in the operating dual bands, respectively. Errors in processing and the loss of substrate may affect the ideal performance.

## Discussion

In this paper, a novel perturbation approach for implementation of the dual-mode dual-band independently SIW filter is proposed by adding different numbers of perturbation via-holes and changing the lengths of the interference slots. The perturbation via-holes symmetrically on both sides of the vertical bisector of each cavity have effects on the electromagnetic distributions of the TE_101_ and TE_102_ modes. However, the interference slot on the vertical line of each cavity only influences the TE_102_ mode. Thus, the lower passband is independently tuned with a range of 29.1% by combining the two variables, while the independent tunable range of 6.5% to the upper passband can be achieved only by the second variable. Besides, it can also be used as a single passband tunable filter with a wide range of 50.9%. The wide tuning range makes the proposed dual-mode dual-band SIW filter of the modern multiband and multistandard attractive in adaptable and cognitive radio systems.
